# Mandatory treatment for methamphetamine use in Australia

**DOI:** 10.1186/s13011-021-00370-1

**Published:** 2021-04-09

**Authors:** Mathew Coleman, Kelly Ridley, Michael Christmass

**Affiliations:** 1 The Rural Clinical School of Western Australia, The University of Western Australia, 35 Stirling Terrace, Albany, Western Australia 6330; 2 Next Step Drug and Alcohol Service, 32 Moore St, East Perth, Western Australia

**Keywords:** Methamphetamine, Mandatory treatment, Civil commitment, Coercive treatment, Substance use, Involuntary treatment, Australia

## Abstract

**Background:**

In 2016, following a flurry of government inquiries and taskforces including calls for mandatory treatment regimes, the Australian community nominated methamphetamine as the drug most likely to be associated as a problem substance. Mandatory treatment for alcohol and other drug problems in Australia consists of broadly two mechanisms compelling a person into treatment: involuntary treatment or civil commitment regimes; and coercive treatment regimes, usually associated with the criminal justice system. This paper aims to provide a review of the evidence for mandatory treatment regimes for people who use methamphetamines.

**Methods:**

Using a narrative review methodology, a comprehensive literature and citation search was conducted. Five hundred two search results were obtained resulting in 41 papers that had cited works of interest.

**Results:**

Small, but robust results were found with coercive treatment programs in the criminal justice system. The evidence of these programs specifically with methamphetamine use disorders is even less promising. Systematic reviews of mandatory drug treatment regimes have consistently demonstrated limited, if any, benefit for civil commitment programs. Despite the growing popular enthusiasm for mandatory drug treatment programs, significant clinical and ethical challenges arise including determining decision making capacity in people with substance use disorders, the impact of self determination and motivation in drug treatment, current treatment effectiveness, cost effectiveness and unintended treatment harms associated with mandatory programs.

**Conclusion:**

The challenge for legislators, service providers and clinicians when considering mandatory treatment for methamphetamines is to proportionately balance the issue of human rights with effectiveness, safety, range and accessibility of both existing and novel mandatory treatment approaches.

## Introduction

The self-reported use of methamphetamine in Australia has been declining since its peak in 2001 and has continued to significantly decline more recently between 2013 and 2016 [1]. However, the proportion of people who use methamphetamine in the more potent crystalline form (‘Ice’) has more than doubled between 2010 and 2016 [1]. Methamphetamine is a potent central nervous system stimulant that is recognised as a drug of dependence and abuse. In Australia, it is only available in illicit forms and cannot be prescribed by medical practitioners. More frequent use of the drug coupled with increased rates of co-occurring other substance use and poor access to support in primary care has resulted in increased hospital emergency department presentations [2–4].

In recent years media reporting of a methamphetamine ‘epidemic’ with potential for catastrophic outcomes has been accompanied by a flurry of urgent government efforts to intervene including taskforces and special inquiries [5–7]. In 2016 there was a clear shift in the Australian community’s perception of drug harms, with methamphetamine being nominated as the drug most likely to be associated with a “drug problem” [1]. This included an increase in the proportion of the community erroneously nominating methamphetamine as the drug responsible for the most deaths in Australia [1]. This is at odds with the clear position of alcohol and tobacco as the leading causes of drug-related harm in Australia and world-wide [8].

With media and community pressure on government policy makers to respond, it is unsurprising that calls for mandatory drug treatment have intensified [5–7, 9, 10]. Mandatory treatment for alcohol dependence was first introduced in Australia in the 1800s. At that time, legislation embodied the concept that “alcoholism was a disease to be treated rather than a crime to be punished” [11]. In 1900, passage of the Inebriates Act of New South Wales (NSW) provided a framework for confinement and rehabilitation to treat alcohol and drug addiction [12]. However, the realities never met the legislative intention and over the last decade most Australian states have modified their mandatory treatment legislation to accommodate human rights, regulatory and ethical considerations into their treatment programs [13].

The current pressure to revisit mandatory treatment in response to changing community attitudes carries a number of potential risks. These include the implementation of treatment models that are poorly evidence-based, may not provide the outcomes desired, may not be provided to people in the most need and are not cost effective. There is an additional risk of diverting limited resources away from more established, effective, accessible and voluntary interventions. Internationally, there has been a trend away from mandatory forms of drug treatment [14]. This paper provides a narrative review of mandatory treatment, the evidence of its effectiveness and provides a discussion of the complex issues associated with mandatory drug treatment in the context of methamphetamine use in Australia.

## Methods

### Sources and selection criteria for the narrative review

A comprehensive literature search was conducted utilising the Medline, Pubmed, PsycInfo, Austhealth, Google Scholar and EMBASE databases. Search terms included “methamphetamine”, “treatment”, “withdrawal”, “pharmacotherapy”, “review”, “involuntary treatment”, “civil commitment”, “involuntary drug and alcohol treatment” and “coercive treatment”. Five hundred two search results were obtained. Publications focussing on child and adolescent populations were excluded for the purpose of focussing the scope of the review on adults (> 18 years of age), as persons who are presumed by law to have capacity to consent (or not) to treatment. Citation searches were conducted in the Web of Science and Scopus databases resulting in 41 papers that had cited works of interest and used for the purposes of this narrative review.

## Results and discussion

### Mandatory treatment regimes

Mandatory treatment regimes broadly involve one of two mechanisms compelling a person into treatment. The first is involuntary or compulsory treatment regimes (or civil commitment) whereby the individual has no determination or say in the matter. The second is referred to as coerced treatment, where individuals must choose between drug treatment or an alternative, for example legal sanction or a criminal justice outcome [13]. See Table 1.

**Table 1:** Coercive Treatment vs Involuntary Treatment Characteristics

	Coercive Treatment	Involuntary Treatment
Definition	Treatment provided to individuals as an alternative to a less desirous outcome.	Short term civil commitment using specifically drafted legislation to treat people with substance dependence against their will in cases where there is imminent risk as a result of substance use.
Examples	Drug Court to avoid incarceration; engagement with AOD services to avoid removal of children by Child Protection Services.	Short term, hospital based treatment for people at imminent risk in the short term as a result of their substance use.
Patient’s consent required?	Yes, patients must choose to engage in treatment and must have the capacity to do so.	No, patients are detained and admitted involuntarily and must be determined to have their capacity to consent undermined by substance use.
Is a substance dependence required?	No	Yes
Treatment setting	Community or residential	Inpatient
Program target outcomes	Reduced drug related harm to the individual, but also the community, family and/or employer	Reduce the immediate harm to the individual, usually by reducing risk of imminent physical risk.
Referring agency	Variable: justice, employer or industrial regulatory authorities, child protection, licencing boards	Health professionals
Australian States and Territories with current programs	All	VIC, NSW, TAS
Level of evidence	2A^a^	4^a^
Summary of Evidence available	Variable result depending on characteristics of the treatment program, with most evidence coming from programs associated with criminal justice system. Some reduced use and better treatment engagement while in the program. Outcomes best with longer periods of engagement, particularly if combined with social supports. Cost effective interventions [13].	Evidence limited, particularly relating to long term outcomes. Models from Australia and internationally have been unable to show they achieved their aims in reducing long term harm, while being costly to provide [13].
Level of evidence for methamphetamine	3B^a^	none

^a^Based on scores using the critical appraisal too from the Centre for Evidence Based Medicine system of grading evidence [15]

Victoria, NSW and Tasmania are the only Australian states with civil commitment regimes currently. The Northern Territory recently disbanded its own version targeting alcohol dependence and public intoxication [13]. Internationally, 90 countries are reported to have some form of compulsory commitment regimes, with the majority having laws supporting coercive rather than civil commitment programs [16]. Internationally, civil commitment regimes have declined in number, whilst coercive criminal justice programs have increased substantially [14].

#### ‘Involuntary treatment’ and ‘civil commitment’

Involuntary treatment regimes, also referred to as civil commitment, are the only pathway into mandatory treatment for people with drug or alcohol problems outside of the criminal justice system in Australia. Civil commitment interventions are generally short term (7–28 days) and explicitly seek to reduce the immediate and significant harms associated with substance use [13]. These programs are provided in an inpatient setting, and are therefore abstinence based [17]. Contemporary legislation underpinning involuntary treatment regimes utilise many of the safe guards for patient autonomy afforded by civil commitment programs established in the treatment of mental illnesses. This includes legislation and resourcing for administrative or legal advocacy or counsel, review and appeal processes, and statutory oversight and governance [18–20].

#### ‘Coercive treatment regimes’

In Australia, coerced acceptance of treatment exists along a continuum from social control through to more readily recognized and regulated regimes. Broadly, coercive enforcement of treatment may include individuals under the supervision of child protection services, child custody or family court arrangements, employment or industrial arrangements, licensing/certification regulations or for people in receipt of social welfare [21]. The most widely researched forms include court-mandated Community Based Orders, Drug Courts, and prison-based treatment regimes [13, 17]. These interventions seek to reduce drug use and associated reoffending, and are only available to those individuals already in the criminal justice system and where programs and resources are available. In some jurisdictions this means geographical (eg rural or remote communities) and offending variables (eg assault or violent offences) can significantly impact on program availability or outcomes. For the purpose of this paper, coercive treatment will be restricted to this later category.

### Mandatory treatment effectiveness

#### ‘Involuntary treatment’ and ‘civil commitment’

Systematic reviews of mandatory drug treatment regimes have consistently demonstrated limited, if any, benefit from civil commitment programs [13, 16, 17]. At best, research on the efficacy for civil commitment regimes is inconclusive and inconsistent with multiple problems cited in the literature [16]. These include poor research methodology, inconsistency across programs and outcome variables, low numbers within trials and loss of participants to follow-up [15].

Initial preliminary evaluations of the civil commitment regime used in NSW (Involuntary Drug and Alcohol Treatment Act 2007) suggested that benefits experienced in the trial may have arisen on the basis of available treatment rather than the mandatory component of care [10]. Increased access to assertive medical, nursing and social care with enhanced follow up and individualised resourcing may account for the positive findings used to support the involuntary nature of the program. Enhanced resourcing compared with voluntary ‘treatment as usual’ may point to issues relating to system wide under-resourcing and funding, rather than biases in the research or effectiveness of involuntary treatment regimes. Furthermore ‘retention in treatment’ whilst under a civil commitment order is a questionable outcome measure by the inherent nature of the enforced treatment. This finding is supported in studies showing legal mandates were positively related to treatment retention but unrelated to treatment engagement [22].

#### ‘Coercive treatment regimes’

In contrast to civil commitment regimes, there is greater volume and robustness to the research undertaken regarding coercive drug treatment programs particularly in the criminal justice system. Nonetheless, drawing conclusions about the effectiveness between programs remains difficult [17, 23]. Small but ambiguous benefits on drug use and criminal recidivism have been demonstrated across some studies [13, 17] with some evidence indicating a cost effectiveness to Court mandated and Drug Court programs in reducing reoffending and hospitalisations relating to drug use [13, 24]. Compulsory prison-based programs have been shown to be a very expensive approach with minimum impact on overall recidivism and drug use [25].

### Mandatory treatment for methamphetamine

With respect to methamphetamine use disorders specifically, there is limited data on the outcomes of mandatory treatment. Studies focusing on coercive treatment in the criminal justice and parents affected child protection systems indicate moderate benefits, similar to voluntary treatment programs [26, 27]. In the United States, 30% of individuals coercively treated through a criminal justice program remained abstinent from methamphetamine use at 24 months [26]. Unsurprisingly, longer periods of intervention and resourcing were positively correlated with better outcomes, consistent with findings in voluntary treatment trials [26]. To our knowledge, no studies have been published regarding efficacy of civil commitment regimes in methamphetamine use disorders.

### Clinical practice and ethical issues in mandatory treatment for methamphetamine

Despite the growing enthusiasm for the development of mandatory treatment regimes for methamphetamine and other drugs, there remains significant clinical and ethical challenges with enforced treatment. It has been argued that a number of responsibilities and considerations are required to legally and ethically justify mandated interventions including: safeguards that the rights of individuals are protected by due process; that enforced treatment is humane and also effective [26]. We consider a number of additional issues specifically in relation to methamphetamines.

#### Capacity determination

According to Australian legal and ethical standards in health care, it is a fundamental principle that treatments cannot be imposed upon an adult who has the capacity to make the decision whether to accept or decline treatment. Consequently, involuntary treatment can only be justifiable when: a person’s dependence has seriously impaired their capacity to make choices about ongoing substance use and personal welfare; care and treatment is necessary to protect the person from significant harm; no other less restrictive means are reasonably available for caring for the person; the person is likely to benefit from the treatment; and the person has refused less restrictive treatment [16].

Assessing treatment decision making capacity in the context of methamphetamine use, dependence, withdrawal and recovery is a clinically vexed and complex process. Methamphetamine use can cause mild to moderate cognitive impairment of variable duration in some, but not all, people who use methamphetamine [28]. Recovery of decision making capacity in the context of even short periods of abstinence may prohibit the use of longer periods of involuntary treatment [29]. Removing existing standards of assessing capacity when considering involuntary treatment would place people who use substance at odds with contemporary mental health principles and legislation. This would effectively create a two tiered system of rights and safeguards depending on the presenting problem rather than on values, principles and universally accepted human rights.

#### Motivation and self determination

Fundamental to much of the research in the addiction field is recognising the importance and centrality of self determination and internal motivation as essential factors for effective treatment and outcomes. Autonomous motivation at the commencement of treatment has been shown to be associated with increased retention in treatment, lower rates of in-treatment drug use, and longer periods of abstinence after treatment completion [30]. Intuitively, anecdotally, and in some limited studies, autonomous motivation is reported as significantly lower in involuntary versus voluntary treatment programs [17]. However it has been argued that external motivators, including criminal justice system coercion, may lead to increased internal motivation or interact with internal motivators to produce positive outcomes [26, 30].

Other potential unintended negative effects of mandatory treatment programs include undermining existing harm reduction strategies and voluntary interventions. This has been noted in some Southeast Asian countries where the use of punitive intervention strategies in compulsory treatment programs seems at odds with other attempts to reduce the harms associated with drug use [17].

### Treatment effectiveness

Mandatory treatment can only be justified where the person is likely to benefit from the treatment, and the treatment is readily accessible. Any discussion of mandatory treatment for methamphetamine use disorder must therefore consider the effectiveness of treatments currently available for both methamphetamine withdrawal and relapse prevention. Limited data exists to define a standard trajectory of methamphetamine withdrawal, commonly including depression, agitation and fatigue [31] and there are no medications licensed for use in methamphetamine withdrawal in Australia. Some studies suggest a biphasic nature to a methamphetamine withdrawal, with an acute phase lasting 7–10 days, and subacute period lasting a further 2 weeks [32]. Other reports indicate most symptoms resolve after 14–21 days [33, 34], however cognitive recovery for some may require an extended period beyond 6 months [29]. Evidence for pharmacotherapies in withdrawal are mixed [35], whilst there is no evidence to date for the use of psychosocial interventions during withdrawal [31].

With respect to relapse prevention, a recent review including 49 studies of 20 potential pharmacotherapies showed no pharmacological agent has consistently demonstrated effectiveness in treating methamphetamine use disorder [36]. Studies of medication approaches typically show poor treatment retention rates between 40 and 50% and there is a general paucity of randomised control trials (RCT) [36]. Dexamphetamine, modafinil, bupropion, naltrexone and methylphenidate are thought to hold some promise [36]. Similarly, mirtazapine, topiramate, fluoxetine, risperidone, buprenorphine, varenicline have also been used, but without controlled RCT evaluation. Evidence of benefit is even more limited for other medications studied in methamphetamine dependence. Of particular note, is the lack of any agonist therapy (equivalent to methadone for opioid addiction) for methamphetamine dependence [35, 36]. It is also important to recognise some pharmacotherapies (e.g. aripiprazole) have the potential for harm [36]. More evidence of benefit is needed, but we also lack data on optimal patient selection and the harms associated pharmacotherapeutic options.

Non-pharmacological approaches remain first line treatment options for relapse prevention in methamphetamine use disorder [35, 37]. Psychosocial interventions include the Matrix Model (MM) [38], Cognitive Behavioural Therapy (CBT) [39] and Contingency Management (CM) [40]. A large Australian RCT of 214 people who use amphetamines demonstrated that a brief intervention comprising of motivational interviewing and CBT was both feasible and effective compared with controls [39]. The reduction in amphetamine use was also accompanied by significant improvements in poly-substance use, injecting risk-taking behaviour, criminal activity, and psychiatric complications such as depression. However, the study was limited in power and did not actively control for the differing number of sessions between the treatment and control groups [39].

CM is practiced extensively in the United States but not commonly used in Australia. Treatment is based on positive reinforcement through provision of a desired item (e.g. retail voucher) contingent on a specific behaviour (e.g. negative urine drug screen). Results repeatedly demonstrate that contingency management has positive effects on methamphetamine use disorder [40]. MM was developed in response to treatment demand for cocaine use disorder in the USA during the 1980s. This program was centered on 36 individual CBT sessions but has been modified over time [38, 41]. Other components of MM include groups for family education (12 sessions) and social support (4 sessions) as well as individual counselling (4 sessions). A large, multisite comparison of MM and treatment as usual showed higher in-treatment abstinence with superior retention and treatment completion in the former. However there were no differences at 6-month followup [41].

Suboptimal accessibility, lack of pharmacotherapy options and non-specialised focus of most treatment services are barriers to treatment engagement for people who use methamphetamine [42]. Enhanced treatment access for people already significantly marginalised by their methamphetamine use has not been adequately studied or, arguably, received investment and attention in Australia. Improving the quality, accessibility and range of available treatments in traditional and assertive outreach models of care must be considered a priority before the considering of the development and use of existing mandatory treatment regimes [10].

### Treatment harms

Of particular concern is the dearth of studies relating to harms associated with mandatory treatment. Some studies examining patient perspectives regarding involuntary admission show that the majority of patients viewed admission as positive [17]. However this finding must be taken in the context of other work that shows that the majority of individuals who experience substance related problems recover without participating in formal treatment programs [30]. Significant negative unintended consequences of mandatory treatment have also been noted from past regimes, including: Net-widening (exposing people to mandatory treatment beyond the purposes and persons for whom it was intended); creating treatment shortages and displacement of limited resources; and, the discrimination against minority groups, as was observed by the Aboriginal community in the recently disbanded Northern Territory mandatory treatment for alcohol dependence and public intoxication [43].

## Conclusions

The challenges for legislators, clinicians and service providers when considering mandatory treatment for drug use disorders, particularly methamphetamine, is to proportionately balance the issue of human rights with the effectiveness, safety and accessibility of both existing and novel mandatory treatment approaches. Careful consideration to unintended consequences must be made in the context of the limited evidence base for mandatory treatment regimes for methamphetamine dependence and related problems. Access, motivation to engage, duration, and acceptability of treatment interventions appear to be important contributors to all mandatory regimes. Coercion based criminal justice programs currently provide small and possibly cost effective options for mandatory treatment whist civil commitment or involuntary treatment is unlikely to be a viable response to increasing alarm and concern in Australia around methamphetamine use.

## Data Availability

Not applicable.

## Abbreviations

NSW: New South Wales
RCT: Randomised control trials
MM: Matrix model
CBT: Cognitive behaviour therapy
CM: Contingency management
